# The clinical approach to diagnosing peri-procedural myocardial infarction after percutaneous coronary interventions according to the fourth universal definition of myocardial infarction – from the study group on biomarkers of the European Society of Cardiology (ESC) Association for Acute CardioVascular Care (ACVC)

**DOI:** 10.1080/1354750X.2022.2055792

**Published:** 2022-05-26

**Authors:** Johannes Mair, Allan Jaffe, Bertil Lindahl, Nicholas Mills, Martin Möckel, Louise Cullen, Evangelos Giannitsis, Ola Hammarsten, Kurt Huber, Konstantin Krychtiuk, Christian Mueller, Kristian Thygesen

**Affiliations:** a Department of Internal Medicine III – Cardiology and Angiology, Heart Center, Medical University Innsbruck, Innsbruck, Austria; b Mayo Clinic and Medical School, Rochester, MN, USA; c Department of Medical Sciences, Uppsala University and Uppsala Clinical Research Center, Uppsala University, Uppsala, Sweden; d University/BHF Centre for Cardiovascular Science and Usher Institute, University of Edinburgh, Edinburgh, UK; e Division of Emergency Medicine and Department of Cardiology, Charité- Universitätsmedizin Berlin, Berlin, Germany; f Emergency and Trauma Center, Royal Brisbane and Women`s Hospital, University of Queensland, Queensland, Australia; g Medizinische Klinik III, Department of Cardiology, University of Heidelberg, Heidelberg, Germany; h Department of Clinical Chemistry and Transfusion Medicine, University of Gothenburg, Gothenburg, Sweden; i 3rd Department of Medicine, Cardiology and Intensive Care Medicine, Wilhelminen Hospital, and Sigmund Freud University Medical School, Vienna, Austria; j Department of Internal Medicine II, Medical University of Vienna, Vienna, Austria; k Department of Cardiology and Cardiovascular Research Institute Basel, University Hospital Basel, University of Basel, Basel, Switzerland; l Department of Cardiology, Aarhus University Hospital, Aarhus, Denmark

**Keywords:** Percutaneous coronary intervention, myocardial infarction, universal definition of myocardial infarction, myocardial injury, cardiac troponin

## Abstract

**Purpose:**

This review intends to illustrate basic principles on how to apply the Fourth Universal Definition of Myocardial Infarction (UDMI) for the diagnosis of peri-procedural myocardial infarction (MI) after percutaneous coronary interventions (PCI) in clinical practice.

**Methods and Results:**

Review of routine case-based events. Increases in cardiac troponin (cTn) concentrations are common after elective PCI in patients with chronic coronary syndrome (CCS). Peri-procedural PCI-related MI (type 4a MI) in CCS patients should be diagnosed in cases of major peri-procedural acute myocardial injury indicated by an increase in cTn concentrations of >5-times the 99th percentile upper reference limit (URL) together with evidence of new peri-procedural myocardial ischaemia as demonstrated by electrocardiography (ECG), imaging, or flow-limiting peri-procedural complications in coronary angiography. Measurement of cTn baseline concentrations before elective PCI is useful. In patients presenting with acute MI undergoing PCI, peri-procedural increases in cTn concentrations are usually due to their index presentation and not PCI-related, apart from obvious major peri-procedural complications, such as persistent occlusion of a large side branch or no-reflow after stent implantation.

**Conclusion:**

The distinction between type 4a MI, PCI-related acute myocardial injury, and chronic myocardial injury can be challenging in individuals undergoing PCI. Careful integration of all available clinical data is essential for correct classification.

## Introduction

The appropriate criteria for diagnosing peri-procedural, PCI-related myocardial infarction (MI) have been controversial for years (Cutlip 2020). This issue was extensively discussed when preparing the consensus documents for the Universal Definition of Myocardial Infarction (UDMI) (Thygesen and Jaffe 2018, Thygesen *et al.* 2019). That document emphasised that the term MI should be applied to patients with acute myocardial injury as defined by a changing pattern of cardiac troponin (cTn) concentrations with at least one value above the 99th percentile upper reference limit [URL] who manifested features of acute myocardial ischaemia by at least one of the following: symptoms of acute myocardial ischaemia, ECG, non-invasive imaging, or invasive coronary angiography.

These criteria were developed from a diagnostic perspective without regard for prognostic considerations. Interventional cardiologists, however, wanted more prognostic guidance so they could be assured that – whatever criteria were used – the diagnosis of peri-procedural MI would identify patients with adverse outcomes. Thus, the Task Force of the Fourth UDMI revised the diagnostic criteria for percutaneous coronary intervention (PCI)-related MI (type 4a MI). They recommended a cTn increase >5-times the 99th percentile URL as the decision limit in patients with normal baseline values or a** **>** **20% rise in cTn together with an increase >5-times the 99th percentile URL if the baseline values are elevated. In addition, they require features of acute myocardial ischaemia as evidenced by new ECG changes, imaging demonstrating new loss of viable myocardium, or complications resulting in impaired coronary flow during PCI (Thygesen *et al.* 2019). PCI-related MI also includes complications of previously implanted stents, stent thrombosis (type 4** **b MI) or in-stent re-stenosis (type 4c MI) (Thygesen *et al.* 2019).

In practice it may be challenging to differentiate peri-procedural acute myocardial injury from type 4a MI. Overt PCI complications seen in coronary angiography are not always associated with significant increases in cTn post-PCI, and elevated cTn concentrations may be found in patients without overt angiographic complications or even after diagnostic coronary angiography without PCI (Uetani *et al.* 2010, Lee *et al.* 2015, Abu Sharar *et al.* 2016, Abu Sharar *et al.* 2020, Mizuno *et al.* 2020, Zhou *et al.* 2020, Arnadottir *et al.* 2021). Increased cTn concentrations after elective PCI do not necessarily indicate procedural related myocardial injury, because baseline, pre-procedural cTn concentrations may already be increased in patients with more extensive and complex coronary artery disease or comorbidities (Uetani *et al.* 2010, Lee *et al.* 2015, Abu Sharar *et al.* 2020, Mizuno *et al.* 2020, Zhou *et al.* 2020). In patients undergoing urgent PCI for acute MI the situation is even more challenging. Apart from obvious major peri-procedural complications, increases in cTn concentrations are usually due to the index presentation and not the procedure itself. Thus, facing these difficulties in everyday clinical practice, we present routine cases highlighting key features that support the diagnosis of PCI-related MI. As with most clinical situations, the correct diagnosis requires clinical judgement and systematic evaluation.

## Presentation of the cases

### Case 1: A 60-year-old male patient with high-risk non-ST segment elevation MI (non-STEMI) referred for acute PCI

**Figure 1.**
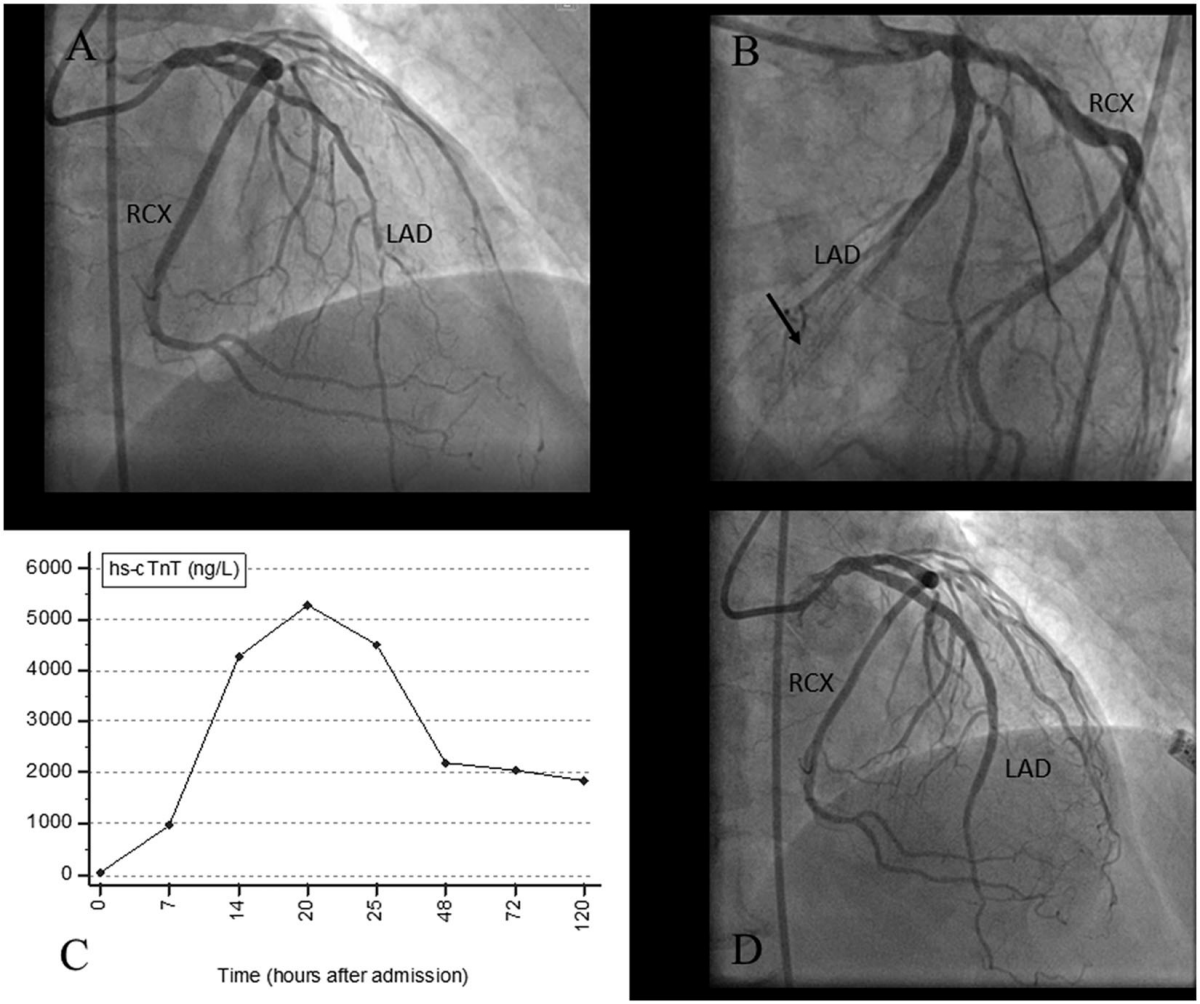
Therapy-refractory no-reflow phenomenon in a patient with anterior wall non-STEMI after stent implantation during acute PCI. Multiple, subtotal stenoses of the LAD as the infarct-related vessel are shown in A, the treatment refractory no-reflow phenomenon after stent implantation is marked by an arrow in B. hs-cTnT release after acute PCI was consistent with type 4a MI (C). A control coronary angiography 3 months later showed normal flow in the LAD without significant stenoses of the implanted stents. hs-cTnT: high-sensitivity cardiac troponin T; PCI: percutaneous coronary intervention; CAG: coronary angiography; LAD: left anterior descending; RCX: left circumflex.

**Figure 2.**
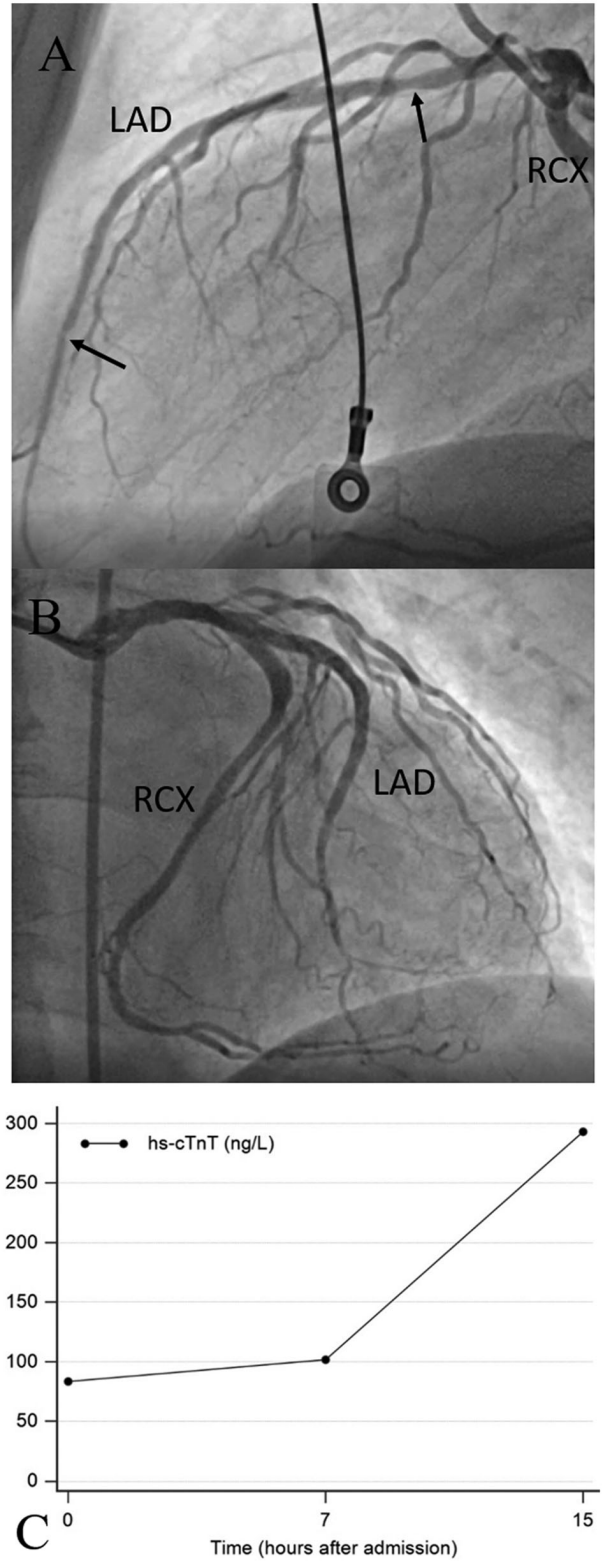
A case of type 4c myocardial infarction. This patient presented with a type 1 non-STEMI 13 month after the initial complex PCI (see Figure 1). The in-stent re-stenoses of the LAD (A, marked with arrows) were successfully treated by balloon dilatation with non-compliant and drug eluting balloons (B). PCI was complicated by temporary slow flow in the LAD, which could be rapidly managed. The peri-procedural hs-cTnT concentration time course in this patient after transfer is shown in C. The contribution of temporary slow flow as a minor complication to hs-cTn release following acute PCI in non-STEMI is difficult to appraise. hs-cTnT: high-sensitivity cardiac troponin T; PCI: percutaneous coronary intervention; CAG: coronary angiography; LAD: left anterior descending; STEMI: ST segment elevation myocardial infarction; RCX: left circumflex.

This patient presented with typical angina to a local hospital. The admission ECG showed ST segment depression in V_2_–V_4_ and an increased hs-cTnT concentration of 40** **ng/L (99th percentile URL ≤14** **ng/L) on admission. Because of ongoing symptoms despite medical therapy, he was transferred by helicopter for acute PCI to the tertiary care centre. Coronary angiography revealed a subtotal occlusion of the left anterior descending (LAD) coronary artery as the culprit lesion (Figure 1(A)). After stent implantation no-reflow occurred that was resistant to tirofiban, intracoronary thrombus aspiration, adenosine, and additional stent implantation proximal and distal to the first stent (see Figure 1(B)). The patient developed ST-segment elevation with decompensation. The hs-cTnT concentration time course associated with this complication is shown in Figure 1(C), the peak concentration rose to 5290** **ng/L. At follow-up coronary angiography three months later, the vessel was patent with Thrombolysis in Myocardial Infarction (TIMI) grade 3 flow (Figure 1(D)). At this visit, left ventricular function had recovered with a normal global left ventricular ejection fraction despite hypokinesia at the apex. The patient did well afterwards, but 13** **months after the index event, he again presented with angina at rest to the local hospital with new T wave inversions in V_2_ and V_3_ compared to his previous discharge ECG. The admission hs-cTnT concentration at the local hospital was increased to 17** **ng/L. The patient was transferred to the tertiary care centre for acute PCI. Coronary angiography demonstrated functionally significant distal and proximal in-stent re-stenoses (see Figure 2(A)), which were treated by balloon angioplasty. Prolonged inflations with non-compliant and drug eluting balloons, however, led to temporary slow-flow without ST-T segment changes, which was rapidly resolved by intravenous tirofiban, intracoronary nitroglycerol and adenosine (see Figure 2(B)). The time course of hs-cTnT concentrations after this third event is shown in Figure 2(C). The admission value at the tertiary care centre was 83** **ng/L, the peak cTn concentration before the patient was transferred back to the local hospital was 293** **ng/l 15** **h after PCI (353% increase from baseline before PCI). There were no further events during a 6-months follow-up.

#### Consensus diagnosis

First event: Type 1 non-ST segment elevation MI

Second event: Peri-procedural type 4a MI due to no-reflow after stent implantation during acute PCI

Third event: Type 4c non-ST segment elevation MI due to in-stent restenosis. The criteria of type 4a MI are not met.

#### Key clinical features that support the diagnosis

First event: Initially the criteria of type 1 non-ST segment elevation MI are met (see Supplemental Table 1).

**Table 1.** Fourth Universal Definition of Myocardial Infarction: types and criteria for the diagnosis of percutaneous coronary intervention (PCI) – related myocardial infarctions (type 4 myocardial infarctions).

*Type 4a myocardial infarction:* PCI-related MI within 48** **h from the index procedure. cTn increase >5-times the 99th percentile URL from baseline values within the URL or >20% increase from an elevated baseline to an absolute value > 5-times the URL together with at least one of the following criteria*: New ischaemic ECG changes; Imaging evidence of new loss of viable myocardium that is presumed to be new and consistent with an ischaemic aetiology; Angiographic findings consistent with a procedural flow-limiting complication sufficient to generate significant myocardial ischaemia, e.g. coronary dissection, distal embolisation of plaque material, side-branch occlusion, slow-flow or no-reflow or disruption of collateral flow. *Isolated development of new pathological Q-waves meets the criteria even if cTn values are elevated and rising but less than the listed threshold or autopsy evidence. *Type 4b myocardial infarction (see* Supplemental figure 2*):* Stent/scaffold thrombosis documented by angiography or autopsy using the same diagnostic criteria as for type 1 MI^#^. The following temporal categories are suggested: Acute ≤24** **h from PCI; subacute >24** **h to 30** **days; late >30** **days to 1** **year; and very late >1** **year after stent/scaffold implantation. *Type 4c myocardial infarction (see* Supplemental figure 3*):* Stent restenosis or restenosis following balloon angioplasty in the infarct territory as the only angiographic explanation of a MI using the same criteria as for type 1 MI^#^.

MI: Myocardial infarction; cTn: cardiac troponin; URL: upper reference limit; ECG: electrocardiogram; hs: high-sensitivity.

^#^The criteria for type 1 MI are summarised in Supplemental Table 1.

Second event: Signs of new myocardial ischaemia (ST segment elevation with acute heart failure) during acute PCI and a marked increase in cTn concentrations after acute PCI meet the clinical criteria of type 4a MI caused by therapy-resistant no reflow phenomenon after stent implantation. Although we are comfortable with the diagnosis in this clinical scenario, it is very difficult to define uniform cTn thresholds for the diagnosis of type 4a MI in patients undergoing urgent PCI for type 1 MI. It is not possible to exactly quantify the extent of this additional PCI-related myocardial injury and the initial type 1 MI in this patient. At 3-month follow up, investigations demonstrated normal flow in the infarct-related coronary artery with a normal left ventricular ejection fraction and hypokinesia in the infarct-related territory.

Third Event: The criteria of type 1 non-ST segment MI are met (see Supplemental Table 1), but given the presence of in-stent restenosis, it fulfils the criteria of type 4c MI (see Table 1). On admission to the tertiary care centre there was ongoing myocardial injury as indicated by increasing cTnT concentrations. Additional myocardial injury may have occurred during acute PCI due to transient slow-flow, but in the absence of significant ST segment changes on ECG monitoring the criteria of type 4a MI are not met. It is impossible to reliably differentiate the extent to which this subsequent, moderate, but >20% cTn rise from baseline before acute PCI occurred due to the index MI or as a consequence of prolonged balloon inflations to treat the re-stenotic lesions. This is also true for other minor peri-procedural complications of acute PCI, such as temporary side-branch occlusion (see Supplemental Figure 1).

**Figure 3.**
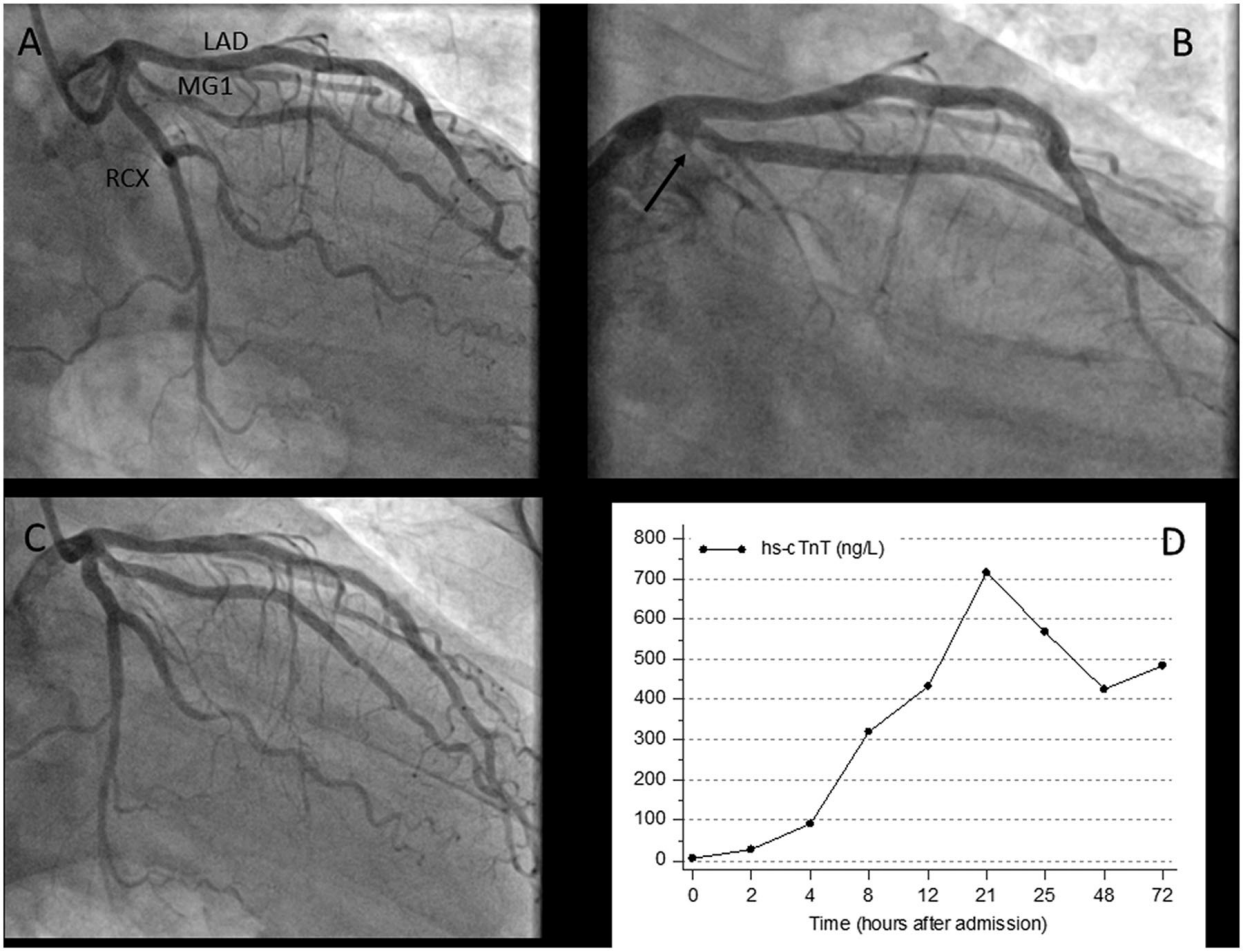
A case of complicated elective PCI. This patient was admitted for elective PCI of a significant stenosis of the first marginal branch of the RCX (A). After stent implantation antegrade and retrograde coronary dissection occurred as a complication with temporary loss of flow in the main branch of the RCX as well (B, arrow). The patient developed angina and ST segment changes in ECG monitoring. This complication could be managed with a good angiographic result (C). The post-procedural hs-cTnT time course after admission to the coronary care unit in this patient is shown in D. The diagnostic criteria of type 4a MI were fulfilled. hs-cTnT: high-sensitivity cardiac troponin T; PCI: percutaneous coronary intervention; RCX: circumflex coronary artery; MI: myocardial infarction; MG1: first marginal branch; LAD: left anterior descending.

### Case 2: A 63-year old male patient scheduled for elective staged PCI of the circumflex coronary artery

This patient underwent acute PCI with stenting of the LAD two months before admission for staged PCI of the circumflex coronary artery. The LAD was found to be patent with no evidence of in-stent restenosis (see Figure 3(A)). After stent implantation in the first marginal branch of circumflex artery antegrade and retrograde coronary dissection occurred as a complication with temporary loss of flow in the marginal branch and main circumflex artery (see Figure 3(B)). The patient developed angina and ST-segment depression on ECG monitoring. This complication was managed with further stent deployment achieving a good angiographic result (see Figure 3(C)). The post-procedural hs-cTnT concentration time course is shown in Figure 3(D). The baseline hs-cTnT value of 6** **ng/L was below the 99th percentile URL (≤14** **ng/L), and the peak value of 717** **ng/L (21** **h after PCI) was 51-times its URL. The patient developed transient new negative T waves in V_3_-V_5_ and new hypokinesis at the basal segment of the inferior wall on echocardiography. The global left ventricular ejection fraction remained normal. There were no events during a 9-months follow-up period.

#### Consensus diagnosis

Type 4a non-ST segment elevation MI

#### Key features that support the diagnosis

1. There was acute peri-procedural myocardial injury, indicated by a significant increase in hs-cTnT concentrations from a normal baseline value, which was substantially greater than the decision limit of 5-times the 99th percentile URL.

2. The patient had consistent evidence of myocardial ischaemia as indicated by ECG changes and a new wall-motion abnormality on echocardiography.

3. Coronary angiography demonstrated a coronary flow limiting complication (dissection).

### Case 3: A 80-year-old male patient referred for elective coronary angiography for angina on exertion

**Figure 4.**
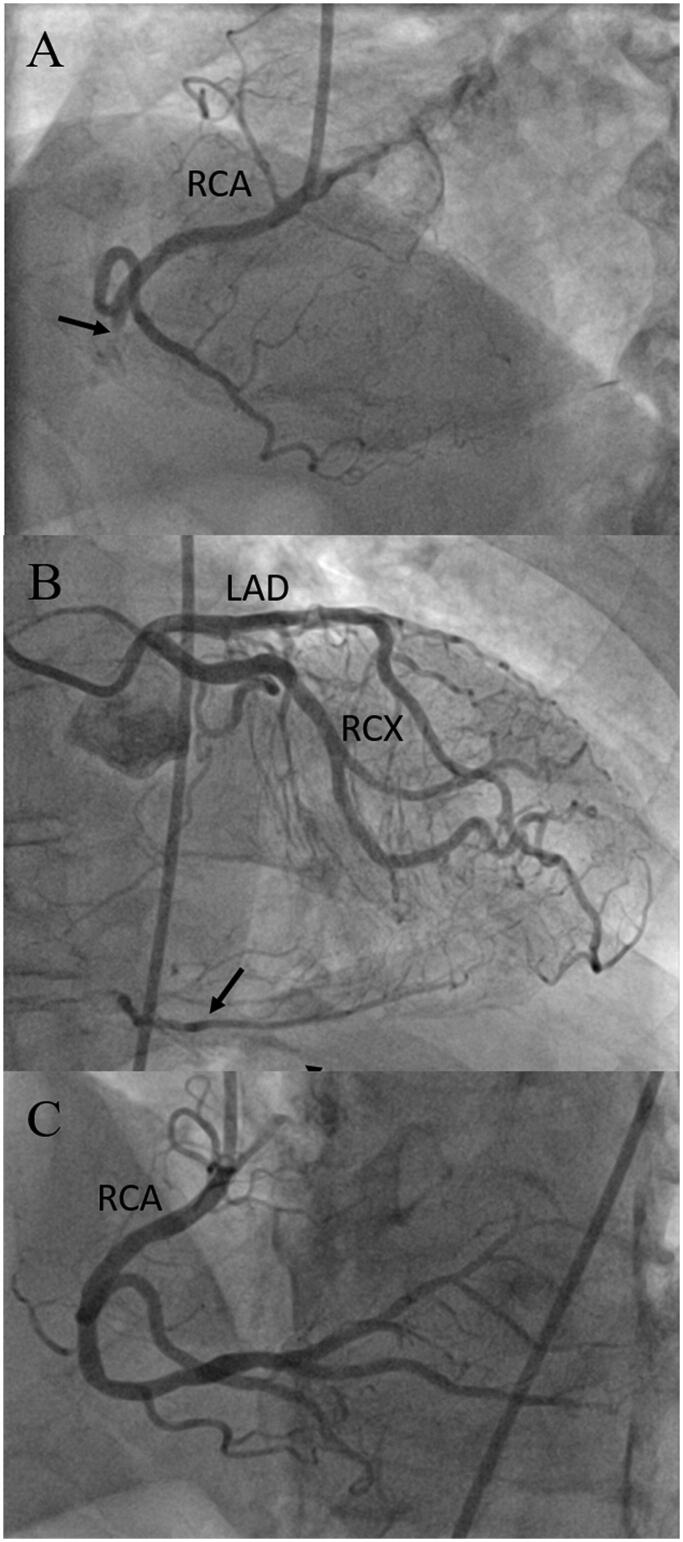
Minor, asymptomatic high-sensitivity cardiac troponin T increase after PCI for chronic total occlusion of the right coronary artery. The RCA was totally occluded in its mid segment (A, marked with arrow), distal RCA was filled via septal collaterals from the LCA (B, marked with arrow), which had no relevant stenosis. The excellent CTO PCI result is shown in C. The patient had no symptoms or signs of myocardial ischaemia during and after PCI. hs-cTnT concentration rose significantly from a baseline value of 6 ng/L (99th percentile URL ≤14 ng/L) to 37 ng/L 12 h after PCI. The asymptomatic patient was discharged on the day after PCI. PCI: percutaneous coronary intervention; RCA: right coronary artery; LCA: left coronary artery; URL: upper reference limit; LAD: left anterior descending; RCX: left circumflex.

This patient complained of angina during physical exercise, which started about 4** **months before a planned admission. The resting ECG was normal, but coronary computed tomography angiography showed a short occlusion of the right coronary artery (RCA) with contrast thought to be from left coronary artery collateral flow. Referral to invasive coronary angiography was delayed because of the COVID-19 pandemic. Invasive coronary angiography confirmed chronic total occlusion (CTO) of the RCA, and this vessel was reopened by PCI using an antegrade approach (see Figure 4(a–c). PCI was uneventful with the patient reporting no angina or having ECG changes during or after the procedure, but the hs-cTnT concentration rose from a baseline value of 6** **ng/L (99th percentile URL ≤14** **ng/L) to 37** **ng/L at 12** **h after PCI. The patient was discharged as planned the day after PCI, and there were no events during a 9-months follow-up period.

#### Consensus diagnosis

Peri-procedural, PCI-related myocardial injury with hs-cTn increase <5-times the 99th percentile URL

#### Key clinical features that support the diagnosis

The criteria of type 4a MI are not met. Post-PCI hs-cTnT concentrations did not rise above 5-times the 99th percentile URL, and there was no evidence of new concomitant peri-procedural myocardial ischaemia.

This patient had no events during follow-up, which is in accordance with most recent analyses (Brauer *et al.* 2013, Lo *et al.* 2014, Auguadro *et al.* 2015, Ndrepepa *et al.* 2016, Koskinas *et al.* 2018, Toma *et al.* 2018, Zeitouni *et al.* 2018, Silvain *et al.* 2021) demonstrating that minor increase in hs-cTn concentrations (<5-times the 99th percentile URL) after antegrade CTO PCI is not associated with worse outcome.

### Case 4: A 79-year old male patient referred for elective coronary angiography after admission to a local hospital because of acute heart failure

**Figure 5.**
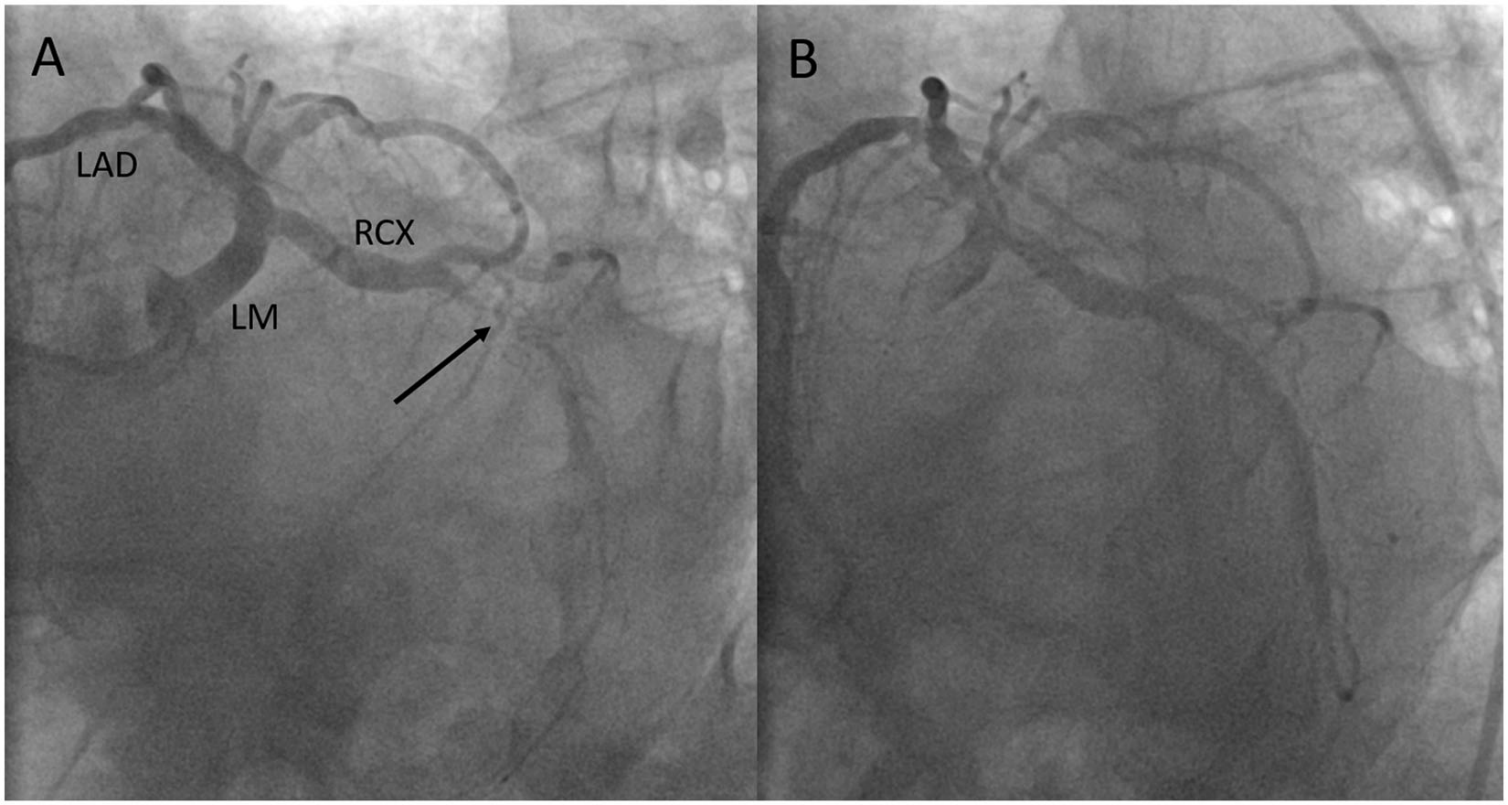
A 79-year old patient referred for coronary angiography after admission to a local hospital because of acute heart failure. This patient presented to a local hospital with dyspnoea and signs of acute heart failure. He had a history of an inferior wall MI with reopening of the circumflex artery and stent implantation 10 years before. Elective coronary angiography revealed a subtotal stenosis of the previously implanted stent (A), which could be reopened and two drug-eluting stents were implanted (B). The hs-cTnT baseline concentration was 19 ng/L and the post-PCI concentration the day after PCI was 22 ng/L, i.e. a change within 20% of the baseline value. PCI: percutaneous coronary intervention; hs-cTnT: high-sensitivity troponin T; LM: left main; LAD: left anterior descending; RCX: left circumflex; MI: myocardial infarction.

This patient with CCS presented acutely to a local hospital with symptoms and signs of acute heart failure. The ECG showed persistent atrial fibrillation with a bifascicular block (complete right bundle branch block with left anterior hemiblock) with non-specific ST-segment and T wave changes. Initially the left ventricular function was severely depressed with severe mitral valve regurgitation. The patient had a prior history of inferior type 1 MI with reopening of the circumflex artery and stent implantation 10** **years before. The admission hs-cTnT concentration was 20** **ng/L [99th percentile URL ≤14** **ng/L] but without significant change >20% on serial testing (22** **ng/L). After acute heart failure management, the patient was admitted to a tertiary care centre for elective coronary angiography to exclude progression of coronary artery disease. The admission hs-cTnT value at this hospital was 19** **ng/L, and the N-terminal pro B-type natriuretic peptide concentrations was markedly increased at 3169** **ng/L. Left ventricular systolic function was then moderately impaired, and there was also some improvement in the severity of mitral valve regurgitation to moderate degree. The angiogram revealed a subtotal occlusion of the previously implanted stent (Figure 5(A)), which could be reopened, and two drug-eluting stents were implanted (Figure 5(B)). The hs-cTnT concentration on the day after the PCI was 22** **ng/l. There were no coronary events during a 12-months follow-up period.

#### Consensus diagnosis

Chronic myocardial injury

#### Key clinical features that support the diagnosis

1. There was mild elevation of hs-cTnT concentrations during the episode of acute heart failure but without evidence for acute myocardial injury on serial testing or other evidence for acute myocardial ischaemia. This scenario does not meet the criteria for an acute MI.
2. There was no significant increase (>20% of the baseline value before PCI) in hs-cTnT concentrations after PCI.
3. There were no dynamic ECG changes or other signs of peri-procedural myocardial ischaemia.
4. The mildly increased hs-cTnT baseline concentration reflects chronic myocardial injury caused by left ventricular dysfunction with increased wall stress perhaps aggravated by obstructive coronary artery disease (Uetani *et al.* 2010, Thygesen *et al.* 2012, Lee *et al.* 2015, Ndrepepa *et al.* 2016, Abu Sharar *et al.* 2020, Mizuno *et al.* 2020, Zhou *et al.* 2020).

## Discussion

The introduction of cTn and in particular hs-cTn assays into daily routine has considerably increased the sensitivity of biomarkers for the detection of myocardial injury including peri-procedural injury in PCI (Talasz *et al.* 1992, Genser *et al.* 1996, Genser *et al.* 1997, Thygesen *et al.* 2012, Thygesen *et al.* 2019). Elective PCI is generally a safe procedure, but peri-procedurally increased cTn and especially increased hs-cTn concentrations are commonly found, particularly in patients with high-risk baseline or procedural features (Uetani *et al.* 2010, Brauer *et al.* 2013, Auguadro *et al.* 2015, Lee *et al.* 2015, Ndrepepa *et al.* 2016, Koskinas *et al.* 2018, Zeitouni *et al.* 2018, Abu Sharar *et al.* 2020, Mizuno *et al.* 2020, Zhou *et al.* 2020, Silvain *et al.* 2021). In contrast to some other descriptions (Moussa *et al.* 2013, Garcia-Garcia *et al.* 2018; see Table 2), the 4th UDMI (Thygesen *et al.* 2019) only lists cTn criteria for the diagnosis of peri-procedural MI, considering that creatine kinase isoenzyme MB (CKMB) does not add useful diagnostic or prognostic information compared with cTn and that CKMB has been phased out by many clinical laboratories as advocated (Jaffe *et al.* 2021). Nonetheless, many interventional cardiologists and the Society for Cardiovascular Angiography and Interventions (SCAI) still prefer CKMB (Moussa *et al.* 2013). Expectantly with CKMB testing, peri-procedural acute myocardial injury is less frequently seen than with cTn (Bertinchant *et al.* 1999, Uetani *et al.* 2010, Thygesen *et al.* 2012, Vranckx *et al.* 2012, Brauer *et al.* 2013, Zeitouni *et al.* 2018, Jaffe *et al.* 2021).

**Table 2.** Criteria suggested for the diagnosis of peri-procedural myocardial infarction in elective PCI.

	CKMB cut-off	cTn baseline ≤URL	cTn baseline > URL	Clinical criteria (≥1 needed)
UDMI Type 4a MI (Thygesen et al. 2019)	n.a.	>5× URL plus clinical criteria	Increase >20% + >5× URL plus clinical criteria	New signs of myocardial ischaemia as evidenced by ECG changes, imaging, or coronary flow-limiting complications
SCAI clinically relevant MI (Moussa et al. 2013)	≥10× URL or ≥5× URL plus clinical criteria	≥70× URL or ≥35× URL plus clinical criteria	≥70× URL or ≥35× URL plus clinical criteria	New Q waves in ≥2 contiguous leads, New persistent LBBB
ARC-2 peri-procedural MI (Garcia-Garcia et al. 2018)	n.a.	≥35× URL	≥35× URL	New Q waves or equivalents, evidence in imaging, coronary flow-limiting complications

PCI: percutaneous coronary intervention; UDMI: Universal Definition of Myocardial Infarction; SCAI: Society for Cardiovascular Angiography and Interventions; ARC: Academic Research Consortium; MI: myocardial infarction; n.a.: not available; URL [99th percentile]: upper reference limit; LBBB: left bundle branch block.

**Figure 6.**
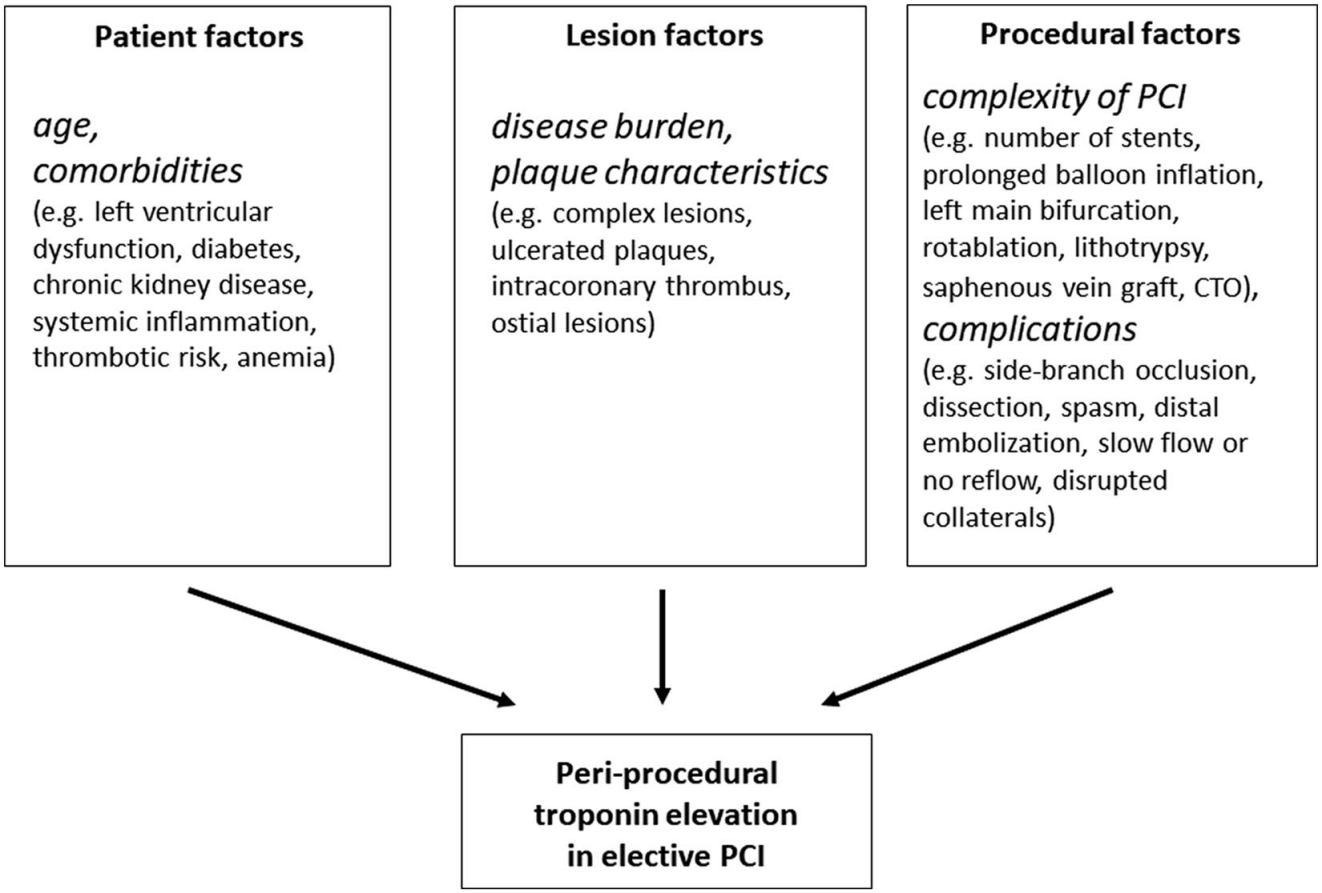
Mechanisms of peri-procedural cardiac troponin increase in elective percutaneous coronary interventions. PCI: percutaneous coronary intervention; CTO: chronic total occlusion.

The pathophysiology of peri-procedural acute myocardial injury in patients undergoing elective PCI is complex (Cuculi *et al.* 2010, Prasad and Herrmann 2011, Bulluck *et al.* 2021), considering that minor peri-procedural myocardial injury (cTn concentrations <5-times the URL) may be related to coronary atherosclerotic burden, unstable plaque morphology, and the complexity of PCI (see Figure 6). In these circumstances most of the prognostic information is contained in the baseline value before PCI (Uetani *et al.* 2010, Lee *et al.* 2015, Ndrepepa *et al.* 2016, Abu Sharar *et al.* 2020, Mizuno *et al.* 2020, Zhou *et al.* 2020). Ndrepepa *et al.* (2016) showed in a large study in CCS patients undergoing elective PCI, that the baseline concentration of hs-cTnT was strongly and independently associated with 3-year all-cause mortality, whereas post-procedural hs-cTnT concentrations did not provide additional prognostic information beyond the baseline concentrations. In patients with urgent PCI for acute MI the situation is complex. Apart from obvious major peri-procedural complications, such as persistent occlusion of a large side branch or no-reflow after stent implantation, fulfilling the coronary angiography criteria of type 4a MI, peri-procedural increases in cTn concentrations are usually due to the index presentation and not PCI-related. As demonstrated by Supplemental case 1, it is very difficult to reliably separate cTnI increase from the initial MI from myocardial injury related to minor PCI related complications.

In clinical practice, many interventional cardiologists currently do not routinely measure cTn in patients undergoing elective PCI. Post-procedural cTn measurements are usually driven by symptoms or peri-procedural complications. We would advise routine measurement of hs-cTn before elective PCI in order to facilitate the interpretation of post-PCI measurements should procedural complications arise. In these circumstances, hs-cTn measurements should be performed just after the procedure in case of ongoing symptoms or a missing baseline hs-cTn concentration, and 3–6** **h later. If there is a significant hs-cTn concentration increase and/or clinical concern the patient can be kept in the hospital for monitoring, and hs-cTn re-testing is suggested on the next day 12–24** **h post-PCI. ECG monitoring and echocardiography are needed to identify myocardial ischaemia and to evaluate its functional consequences.

It has been shown, that type 4a MI is associated with an adverse prognosis after elective PCI (De Labriolle *et al.* 2009, Milani *et al.* 2009, Liou *et al.* 2015, Zeitouni *et al.* 2018, Koskinas *et al.* 2018). Type 4a MI was associated with an about 2-fold higher cardiovascular 1-year event rate and an about three-fold higher, but still low 1-year mortality (3%) in patients with normal hs-cTn baseline concentrations (Zeitouni *et al.* 2018, Koskinas *et al.* 2018). As confirmed by case 2, angiographically visible complications have been reported to usually lead to a marked hs-cTn concentration increase (>35-times the 99th percentile URL) (Koskinas *et al.* 2018).

Recently the term ‘major peri-procedural acute myocardial injury’ has been reintroduced for patients with isolated acute peri-procedural myocardial injury with a cTn increase >5-times the 99th percentile URL after elective PCI not fulfilling the criteria of type 4a MI (Bulluck *et al.* 2021). What is evident from existing studies (see Table 3) and pooled patient-level data analysis and metaanalysis is, that there is a continuous relationship between the extent of peri-procedural myocardial injury and prognosis (Koskinas *et al.* 2018, Zeitouni *et al.* 2018, Li *et al.* 2020, Silvain *et al.* 2021), which is also apparent after cardiac surgery (Devereaux *et al.* 2022). After elective PCI major peri-procedural acute myocardial injury was seen in about 18% of patients and was associated with an about two-fold increased 1-year all cause mortality in patients with a normal cTn baseline value (Silvain *et al.* 2021). A hs-cTnT increase without ancillary signs of myocardial ischaemia, however, was reported to be only a weak predictor of mortality (Koskinas *et al.* 2018). In the complex setting of chronic total occlusion PCI peri-procedural myocardial injury is more common, particularly with retrograde approach (Lo *et al.* 2014, Toma *et al.* 2018). The higher rate of cTnT increase following retrograde procedures did not translate into worse survival, increases ≥50-times the cTn 99th percentile URL within 24** **h, however, were linked with increased 1-year event rate in patients with retrograde CTO PCI as well (Lo *et al.* 2014, Toma *et al.* 2018).

**Table 3.** Prognostic relevance of elective percutaneous coronary intervention-related high-sensitivity cardiac troponin increase.

Study, first author	*n*	Biomarker, manufacturer	Baseline hs-cTn	Blood sampling regimen	Peak hs-cTn cut-off, additionally criteria required?^#^	Endpoint
Koskinas et al. 2018	8140	hs-cTnT, Roche	>URL in 39%	0, 6** **h, daily until discharge	980** **ng/L, no 140** **ng/L, no	1-year cardiac mortality: HR 4.2 HR 1.9 (ROC optimised cut-off)
Ndrepepa et al. 2016	5626	hs-cTnT, Roche	>URL in 38%	0, 6** **h, daily until discharge	Normal baseline value, increase >14** **ng/L, no Elevated baseline value, further increase, no All patients, baseline value, no	3-year mortality HR 2.4 HR 1.09, n.s. HR 1.22, per SD increase
Zanchin et al. 2016	2029	hs-cTnT, Roche	>URL in 26%	0, at least 1× <12** **h post-PCI	14** **ng/L, no baseline Post-PCI	1-year mortality HR 2.1 n.s.
Zhou et al. 2020	1572	hs-cTnT, Roche	< URL in all	0, 16-24** **h post PCI	>14** **ng/L, no	Median 1.5** **year follow-up: mortality, MI rate - n.s. unplanned revascularization – HR 1.4
Zeitouni et al. 2018	1390	hs-cTnT, Roche	<URL in all	0, at least once <48** **h post PCI	70** **ng/L, yes (type 4a MI criteria) 980** **ng, no	1-year CV ischaemic events HR 1.9 HR 3.9
Liou et al. 2015	434	hs-cTnT, Roche	if 0 >URL, stable or declining	0, 12–48** **h post PCI	70** **ng/L or >20% increase if 0 >URL, yes (Type 4a MI criteria)	1-year combined outcome (all-cause mortality, MI, or need for revascularization) HR 7.3, *p*** **=** **0.003
Ferreira et al. 2017	383	hs-cTnI, Abbott	>sex-adjusted URL in 15.5%	0 (in 296 patients), at least 1 × 6–24** **h post-PCI,	Post-PCI: 65** **ng/L (women), 165** **ng/L (men), no	1-year mortality 8.1 vs. 3.1%, *p*** **=** **0.03

Only studies with baseline hs-cTn testing (indicated as 0) are listed. Dedicated studies exclusively on chronic total occlusion PCI are not listed. No large studies on the prognostic significance of hs-cTnI release after elective percutaneous coronary interventions have been published so far.

^#^The URL and cut-off values are listed as applied in the listed studies. 14** **ng/L was the overall 99th percentile URL for the hs-cTnT assay (Roche^®^) in all studies, sex-specific URLs were not used. Ferreira applied sex-specific URLs for the hs-cTnI assay used (Abbott^®^; 33** **ng/L [men] and 13** **ng/L [women]).

*n*: number of patients; hs-cTn: high sensitivity cardiac troponin; PCI: percutaneous coronary intervention; HR: adjusted hazard ratio; URL: upper reference limit; ROC: receiver operating characteristics; n.s.: not significant; SD: standard deviation; MI: myocardial infarction; CV: cardiovascular; vs: versus.

In type 4a MI patients with flow limiting complications, the aim is to achieve an optimal result at the end of the procedure. Additional measures in these patients and the therapeutic consequences of PCI related stand-alone increases in cTn concentrations without accompanying features of myocardial ischaemia or effective pharmacological measures for prevention of peri-procedural myocardial injury are not well defined (Cuculi *et al.* 2010, Vranckx *et al.* 2012). Pharmacotherapy for the secondary prevention of future cardiovascular events should be optimised as recommended by current European Society of Cardiology (ESC) revascularization and CCS guidelines (Neumann *et al.* 2019, Knuuti *et al.* 2020).

*Critical clinical concepts for the interpretation of cardiac troponin or high-sensitivity cardiac troponin concentrations in PCI:*

1. Cardiac troponin (cTn), especially high-sensitivity cardiac troponin (hs-cTn), is the biomarker of choice for the diagnosis of myocardial injury and type 4a MI following PCI.
2. hs-cTn should be measured in all patients before elective PCI. In most circumstances, the knowledge of a baseline value is required to correctly interpret increased hs-cTn concentrations after PCI in patients with symptoms or complications.
3. Except for an acute presentation, baseline hs-cTn concentrations are related to coronary artery disease burden, lesion complexity, and prognosis in patients with CCS.
4. Increased cTn concentrations after elective PCI are common and indicate myocardial injury, which, when in the absence of myocardial ischaemia, do not meet the criteria for type 4a MI.
5. Minor peri-procedural myocardial injury (cTn increase ≤5-times 99th percentile URL) after elective PCI reflects coronary artery disease burden, lesion characteristics, or PCI complexity.
6. The symptomatic benefits of elective PCI are likely to be more important than the presence of isolated myocardial injury with cTn increase ≤5-times 99th percentile URL.
7. Major peri-procedural myocardial injury (cTn increase >5-times 99th percentile URL) has independent prognostic significance; its severity is related to the magnitude of cTn increase.
8. The diagnosis of PCI-related MI requires, apart from acute myocardial injury as defined as a significant increase in cTn concentrations >5-times the 99th percentile URL, the presence of new signs of acute myocardial ischaemia caused by complications of the procedure. The cut-off value of 5-times the 99th percentile URL applies to all cTn assays including hs-cTn assays.
9. In patients undergoing PCI for an acute MI, it is very difficult to differentiate the extent of cTn release due to the initial index event from potential acute peri-procedural myocardial injury or type 4a MI due to PCI complications in the absence of an overt PCI-related flow-limiting complication.

## Supplementary Material

Supplemental Material

## Data Availability

The authors confirm that the data supporting the conclusions referring the presented clinical cases are available within the article [and/or] its supplementary materials. More clinical details on the discussed cases are available upon reasonable request by contacting the corresponding author. Data derived from public domain resources (published literature) is covered by the list of references.
