# Supplementary material for: The clinical approach to diagnosing peri-procedural myocardial infarction after percutaneous coronary interventions according to the fourth universal definition of myocardial infarction – from the study group on biomarkers of the European Society of Cardiology (ESC) Association for Acute CardioVascular Care (ACVC)
Source: Biomarkers. 2022 May 26;27(5):407–17. doi: 10.1080/1354750X.2022.2055792 (PMC9344934; doi:10.1080/1354750X.2022.2055792)
Supplement: Supplemental Material [file IBMK_A_2055792_SM0842.zip › Supplementary Material IBMK 2055792/Supplemental table 1.docx]

Supplemental table 1: Criteria for type 1 MI:

Detection of a rise and/or fall of cTn values with at least one value > the 99^th^ percentile URL with at least one of the following:

Symptoms of acute myocardial ischemia;

New ischemic ECG changes;

Imaging evidence of new loss of viable myocardium or new regional wall motion abnormality in pattern consistent with an ischemic etiology;

Identification of plaque rupture or erosion with coronary thrombus formation by angiography, intracoronary imaging, or autopsy.
