# Supplementary figures and images for: The clinical approach to diagnosing peri-procedural myocardial infarction after percutaneous coronary interventions according to the fourth universal definition of myocardial infarction – from the study group on biomarkers of the European Society of Cardiology (ESC) Association for Acute CardioVascular Care (ACVC)
Source: Biomarkers. 2022 May 26;27(5):407–17. doi: 10.1080/1354750X.2022.2055792 (PMC9344934; doi:10.1080/1354750X.2022.2055792)

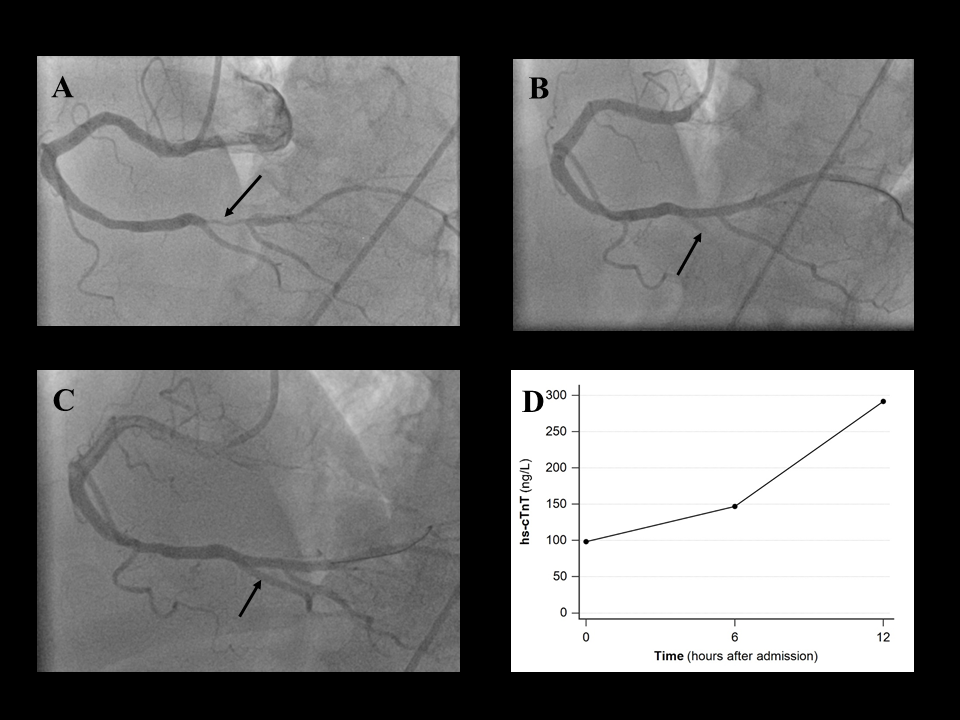

Supplement: Supplemental Material [file IBMK_A_2055792_SM0842.zip › Supplementary Material IBMK 2055792/Supplfigure1.tif]

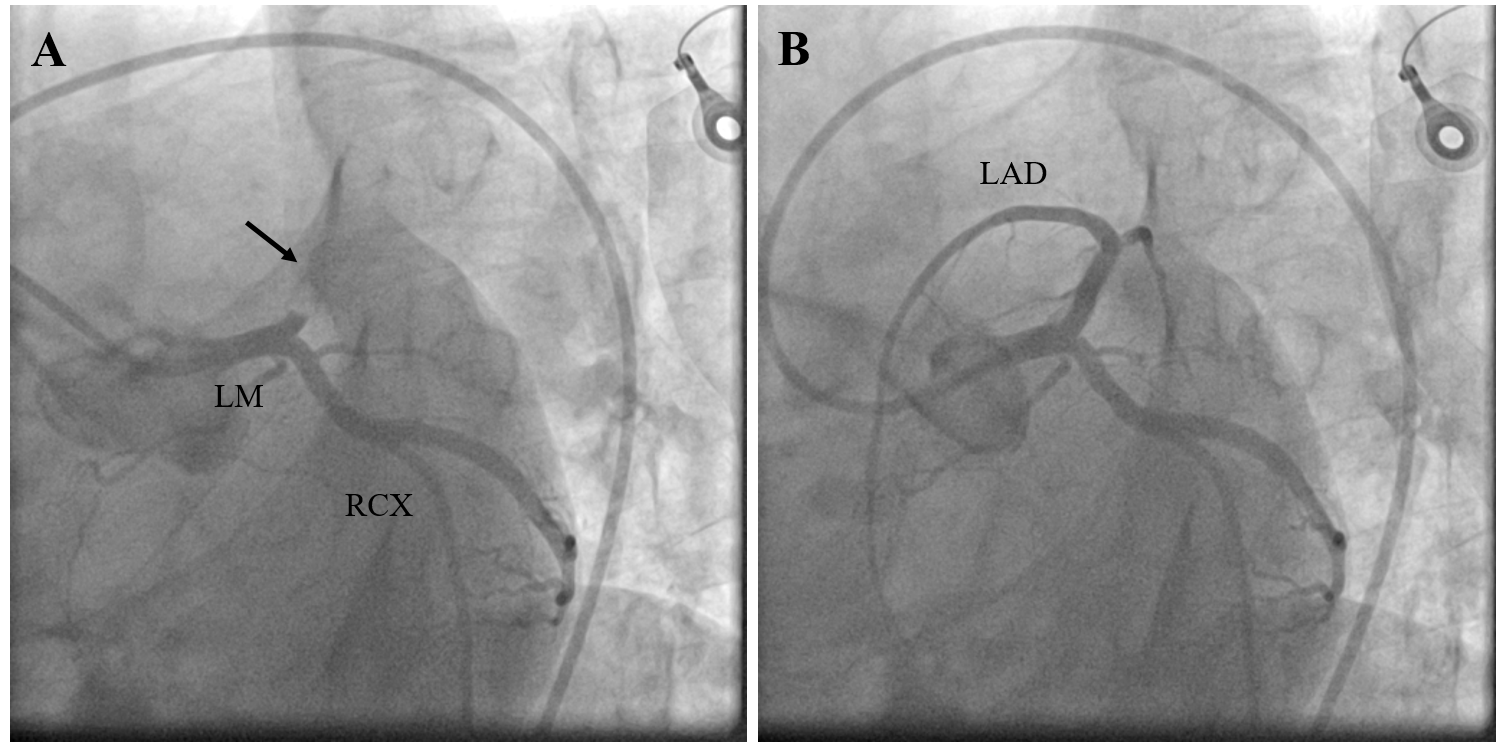

Supplement: Supplemental Material [file IBMK_A_2055792_SM0842.zip › Supplementary Material IBMK 2055792/SupplFigure2.tif]

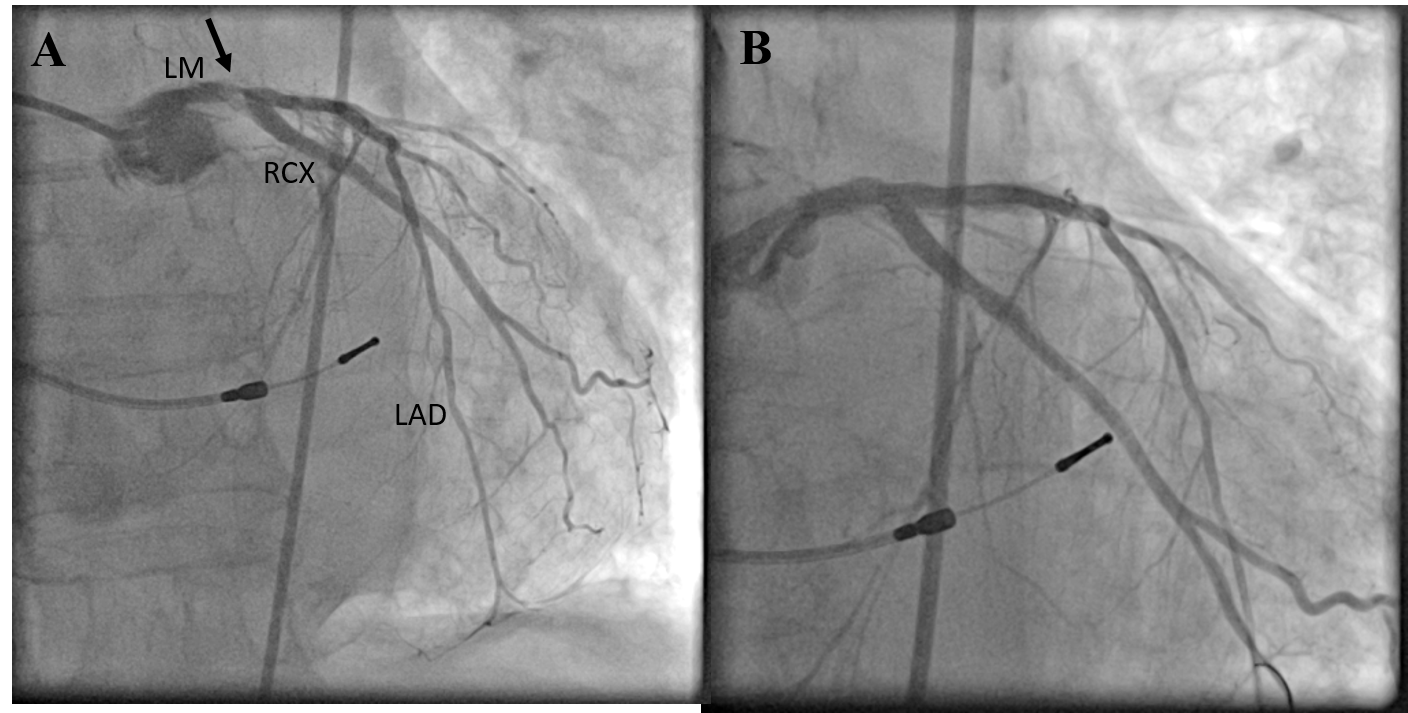

Supplement: Supplemental Material [file IBMK_A_2055792_SM0842.zip › Supplementary Material IBMK 2055792/SupplFigure3.tif]
